# The use of clickers to evaluate radiographer’s knowledge of shoulder images

**DOI:** 10.4102/hsag.v24i0.1053

**Published:** 2019-08-15

**Authors:** Ida-Keshia Sebelego

**Affiliations:** 1 Department of Clinical Sciences, Central University of Technology, Bloemfontein, South Africa

**Keywords:** clickers, criteria, shoulder imaging, knowledge, radiography

## Abstract

**Background:**

Conducting research can be daunting, although applicable methods can facilitate the process. A study was performed at an imaging department pertaining to the routine shoulder projections, namely the anteroposterior (AP) external rotation and lateral-Y (LAT-Y) projections.

**Aim:**

The aim of the study was to determine if radiographers (qualified, supplementary, community service) and student radiographers (second-year diploma, third-year diploma, second-year bachelor) use the radiographic evaluation criteria to evaluate the routine shoulder projections.

**Setting:**

The study was conducted at an imaging department in the Free State province, South Africa.

**Methods:**

Participants had to complete a survey by means of a questionnaire that was compiled in Microsoft Excel and converted to an audience response system known as clickers. The questions addressed aspects of shoulder imaging with regard to positioning, exposure factors and the evaluation of routine shoulder projections. The data were analysed separately using statistics software SAS Version 9.2. Fisher’s exact test was used to determine statistically significant differences between students and radiographers.

**Results:**

More than 80% of students selected the AP (external rotation) X-ray image demonstrating optimal milliamperage per second whereas 43% of radiographers selected the correct image. More than 50% of radiographers and students indicated that a breathing technique and a short exposure time reduce motion during shoulder imaging.

**Conclusion:**

Using clickers eased the process of testing the participants’ knowledge, and the results were available immediately after completion of the test. Clickers can contribute to and expedite the process of data analysis.

## Introduction

It was observed that radiographers (qualified, supplementary, community service and student) have difficulty in obtaining optimal routine shoulder projections, which include the anteroposterior (AP) external rotation and lateral-Y (LAT-Y) projections. The aim of this study was to determine through the use of clickers whether the radiographers at a participating imaging department in the Free State province, South Africa, used the radiographic evaluation criteria of the shoulder to critique routine shoulder projections. The research question was ‘Do the radiographers utilise the radiographic evaluation criteria when critiquing shoulder images?’. Data collection consisted of the use of clickers – also referred to as audience response systems (ARS) and classroom response systems (CRS).

Clickers are a versatile and increasingly popular technology currently used for assessment and surveys in a wide variety of areas, including businesses, conferences and education. With regard to education, in addition to redirecting students’ classroom involvement from passive to active, it delivers real-time feedback to instructors (Gousseau, Sommerfeld & Gooi 2016). Furthermore, the clicker system is convenient to use in classrooms that are constantly growing with regard to student numbers (Tregonning et al. 2012).

Clickers can be used in classroom activities for the assessment of knowledge (Blasco-Arcas et al. 2013). Questions are posed and a number of answer options are offered, from which the participant has to select the correct answer. The results (responses) during discussions are downloaded and saved for record keeping and future use (Martyn 2007).

An important reason why participants engage in clicker activities is the anonymity offered by these devices (Kennedy & Cutts 2005; Martyn 2007; Trees & Jackson 2007). Because of the fact that anonymity increases involvement, participants do not feel pressurised to become involved in clicker activities. The anonymity also creates a safe environment in the sense that participants do not feel humiliated or anxious about giving wrong answers (Martyn 2007; Trees & Jackson 2007). Therefore, using clickers ensures that all participants are involved in the discussion (Martyn 2007; Preszler et al. 2007).

The benefits of using clickers are that it promotes active, collaborative learning and increases student engagement (Blasco-Arcas et al. 2013; Duncan 2005; Lam & Tong 2012; Martyn 2007). It also increases learning motivation (Lam & Tong 2012), class attendance (Duncan 2005) and participants’ interest in the topic and their own learning (Preszler et al. 2007). Another benefit of clickers is that it provides immediate feedback. The feedback is made available to the participants and the facilitator or instructor, who can provide an overview of their understanding of the content under discussion (Blasco-Arcas et al. 2013; Duncan 2005; Kennedy & Cutts 2005; Martyn 2007; Preszler et al. 2007; Trees & Jackson 2007).

In all medical and allied health professions, including radiography, students have to apply their theoretical knowledge in clinical practice. Being actively involved in the learning process will guide them to develop critical thinking skills, especially when interacting with their peers (Trees & Jackson 2007). Critical thinking is imperative, because students need to reason out all their options and reflect on the knowledge that they have on a topic before deciding on an answer or solution. When all the students’ answers are displayed, they can reflect on their peers’ reasoning regarding certain answers, and when using clickers, the correct answer is provided with the necessary explanation (Blasco-Arcas et al. 2013; Kennedy & Cutts 2005).

There is a lot of information on how clickers are used in education, but no information regarding clickers use in research could be found. Hence, there exists a gap in literature regarding the use of clickers to conduct research. Considering the advantages of clickers in education, the researcher used clickers to conduct a research study to evaluate the practical application and theoretical knowledge that radiographers have with regard to the anatomy of the shoulder, the evaluation for optimal positioning and exposure factor selection.

## Methods

A descriptive research design was used to conduct the study. Multiple choice questions (MCQs) from the literature were used in Microsoft Excel to compile a radiographic critique questionnaire (RCQ). The RCQ contained closed-ended questions which required participants to answer either ‘yes’ or ‘no’, or to select the correct answer from a list of options provided (Goddard & Melville 2001). The questions were designed to obtain specific information on how radiographers critiqued shoulder images before they were sent to the radiologist or referring doctor for interpretation, and how they applied their radiographic technique to obtain projections of the shoulder in relation to identifying anatomy, identifying X-ray images demonstrating optimal exposure, and positioning for the AP (external rotation) and LAT-Y shoulder projections. The participants also had to indicate whether they instructed patients to apply a breathing technique during imaging of the shoulder.

The questionnaire from Microsoft Excel was converted to the clicker questionnaire; thus, data were collected by means of a clicker questionnaire. The clicker questionnaire was compiled using the TurningPoint program. The TurningPoint program integrates with PowerPoint to create an interactive and enjoyable presentation. This program also provides the option for producing interactive slides, setting up and running a presentation, and generating reports based on the results. The correct answer in the TurningPoint program was highlighted to assist the statistician when the data had to be analysed to determine the number of participants who selected the correct answer. Other TurningPoint features include participant monitoring and reporting tools. The clicker questionnaire (38 questions) had 10 more questions compared to the questionnaire from Microsoft Excel (28 questions), because five questions from Microsoft Excel had to be subdivided and presented individually for the clicker questionnaire. For example, in question six from the Microsoft Excel questionnaire, the participant had to identify the anatomical structures A to E for an AP external rotation shoulder image. Thus for the clicker questionnaire, question six was subdivided into five questions.

The RCQ on the clicker system was pilot-tested, which enabled the researcher to identify challenges that needed to be addressed before the participants completed the questionnaire. It was noticed that the answers of the pilot participants were anonymous, meaning that their answers were invisible because of a setting in the software. After the pilot study, some settings had to be adapted to ensure that the participants’ answers were visible. The responses of the pilot clicker session were sent to the statistician. The responses did not meet the statistician’s requirements and more settings had to be corrected. The pilot study was extremely valuable in assisting the researcher to address any potential problems related to the clicker session of the main study. The results from the pilot study were not included in the main study.

### Data collection

The TurningPoint receiver was connected to a computer to link all the clickers to the recording software. The participants were requested to switch on the clickers and ensure that they were set to channel 41 to pick up the receiver in order to transmit the answers to the TurningPoint program. Thereafter, the participants were informed that they had to choose the number on the clicker corresponding with the correct answer and then select Enter to transmit the answer to the program. After the process was explained, the clicker session started and the questionnaire was completed in the presence of the researcher. All the radiographers (qualified, supplementary, community service and student radiographers) had 40 minutes to complete the questionnaire. After completion, the responses were saved for record keeping purposes and future use (Martyn 2007) and were exported to Microsoft Excel for analysis.

The anonymity of the participants was protected because each clicker had its own unique number, providing each participant with a unique number. Moreover, the participants did not have to provide their personal information on the questionnaire. In order for the researcher to visualise the answers of the participants after completion of the questionnaire, the anonymous setting had to be disabled prior to the completion of the questionnaire, but did not compromise the anonymity of the participants. The questionnaire responses were saved in the TurningPoint program and could be obtained for possible future use.

The radiography students (second-year diploma, third-year diploma and second-year bachelor students) completed the clicker questionnaire on campus, at a time that did not disrupt their work or studies. The community service, supplementary and qualified radiographers answered the clicker questionnaire in the boardroom of the participating imaging department. Two sessions were arranged at the participating imaging department which were held at 08h00 in the morning. Work-integrated learning co-ordinators and lecturers from an academic institution assisted at the participating imaging department. These aforementioned individuals supervised the students, while the radiographers (qualified, supplementary and community service) completed the questionnaire. Therefore, the sessions did not disrupt the work of the imaging department nor interfere with any other activities of the participants. Both the students and radiographers completed the same clicker questionnaire.

### Data analysis

The manner in which the results (responses) from the clicker questionnaire were saved by the TurningPoint program was deemed satisfactory for data analysis by the statistician. The results were displayed as percentages, and no answers were linked to specific participants. To ensure validity of the results, the statistician analysed the data separately from the data provided by the TurningPoint program.

Further analysis was performed by the statistician using SAS Version 9.2. Descriptive statistics, namely frequencies and percentages, were calculated for categorical data. Means and standard deviations or medians and percentiles were calculated for numerical data. The results of the analysis were displayed as graphs. Fisher’s exact test was used to compare the percentages of the qualified radiographers’ and students’ analytical statistics. A significance level (α) of 0.05 was applied. A *p*-value of ≤ 0.05 indicated a significant difference between the radiographers’ and students’ results.

### Ethical considerations

Approval to conduct the research was obtained from the Ethics Committee of the Faculty of Health Sciences, University of the Free State (ECUFS 100/2015), and the Department of Health of the Free State Province. Further permission was obtained from the Head of Clinical Services and the Director or Head of Department of the participating institution. Additionally, permission was also obtained from the radiography students’ training institution, because they had to complete the questionnaire on the premises of the university.

An information document accompanied the RCQ that was distributed to the participants. This document referred to aspects such as an overview of the purpose of the study, an explanation of what was required of each participant and the contact details of the researcher. A written statement was also included to confirm that participation was voluntary and that the participant could withdraw from the study at any time. The participants signed an informed consent document to partake in the study. The students that participated in the study were not advantaged in any way. Moreover, the students that did not participate in the study were not disadvantaged at all. All the information received from the participants remained anonymous and was available to only the researcher and the supervisors.

All the information collected from the participating imaging department was managed in a strictly professional and confidential manner. Participants were not required to indicate their personal details for the RCQ on the clicker system or identify the hospital where they worked. Therefore, the name of the participating imaging department was not disclosed.

## Results

The RCQ results on the clicker system displayed the data as percentages. Two questions enquired about the demographics of the participants. The sample size of the study was 41 participants, which included 27 (66%) student radiographers, one (2%) supplementary radiographer, one (2%) community service and 12 (30%) qualified radiographers.

The qualified, supplementary and community service radiographers also had to indicate their years of experience, which was asked as an open-ended question. The level of experience ranged between one year and 32 years, with a mean of 18 years’ experience. Five questions from the clicker questionnaire are displayed and discussed in this article to illustrate how clickers were beneficial for the research to collect data.

### Selection of exposure

**FIGURE 1:**
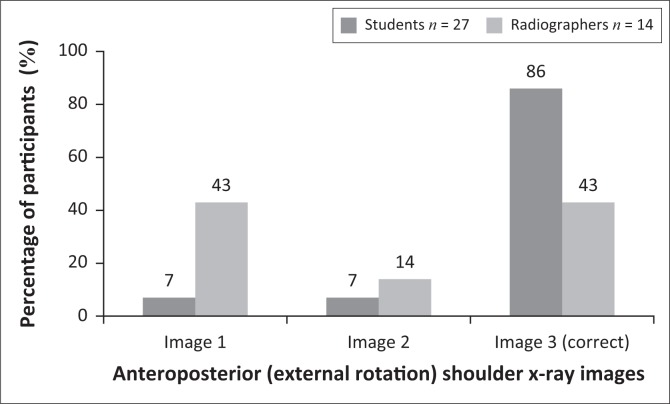
Participants’ answers to the question related to the image showing optimal mAs for anteroposterior (external rotation) projection of the shoulder.

Three X-ray images with different exposure factors were displayed in relation to the AP external rotation shoulder projection images. The participants had to select the X-ray image that optimally demonstrated the milliamperage per second (mAs). The wrong images were selected by 14% of students and 57% of qualified radiographers, as presented in Figure 1. This difference of 43% between the two groups was statistically significant, with *p* = 0.0076.

**FIGURE 2:**
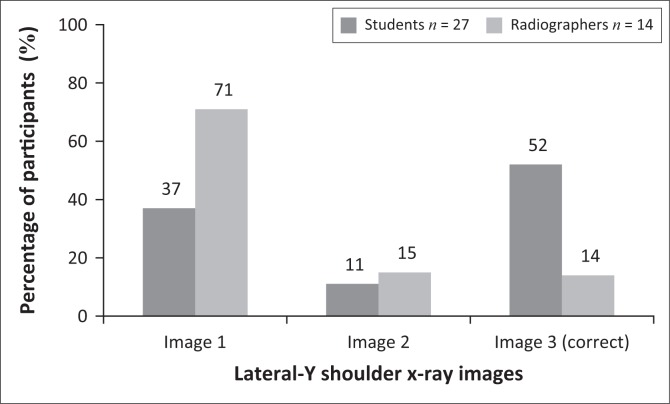
Participants’ answers to the question related to the image showing optimal mAs for lateral-Y projection of the shoulder.

Three LAT-Y shoulder projection images were displayed and required participants to select the image that optimally demonstrated the mAs. Figure 2 shows that 48% of students and 86% of qualified radiographers selected the incorrect answer. Image 3 as seen in Figure 2 was the correct choice, because bony trabecular detail, cortical outlines and soft tissue around the lateral and superior region of the shoulder could be visualised. The difference between the students’ and qualified radiographers’ answers was not significant (*p* = 0.0558).

### Radiographic technique

**FIGURE 3:**
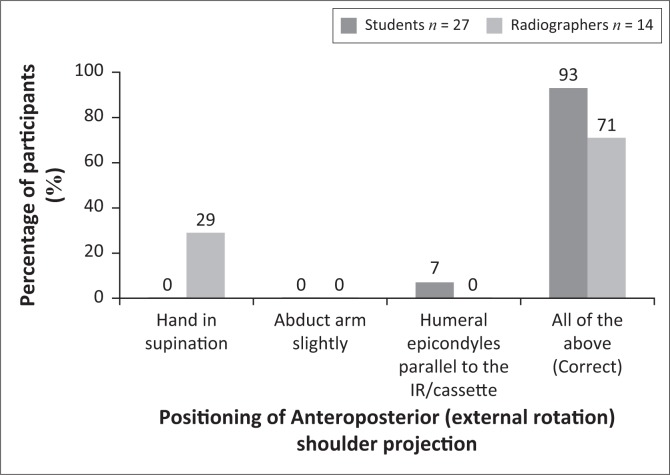
Participants’ answers with regard to the factors applicable to obtain optimal anteroposterior (external rotation) projection.

**FIGURE 4:**
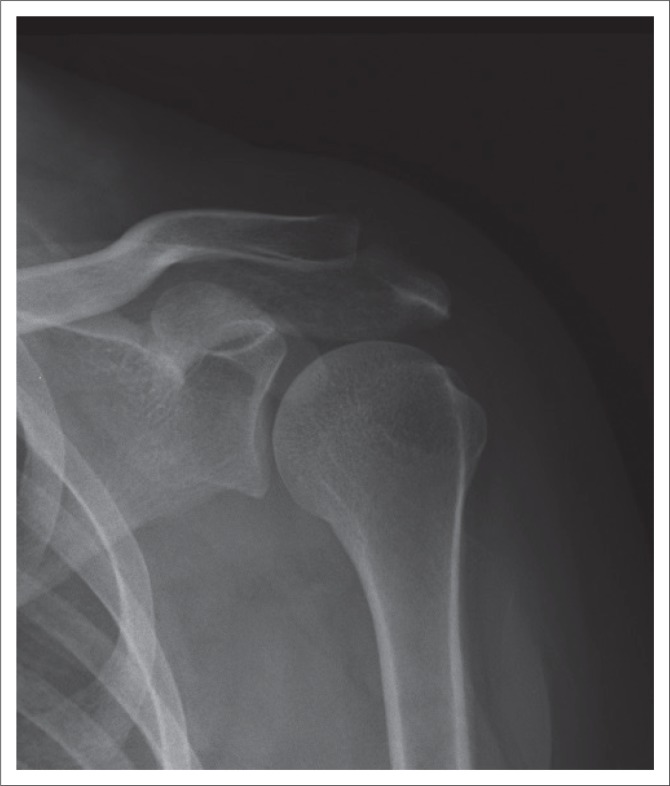
An anteroposterior (external rotation) shoulder image demonstrating correct positioning.

Various factors were listed in a specific question, and the participants had to indicate which of these factors were important to ensure that the AP external rotation shoulder projection was demonstrated optimally. Most of the students (93%) and radiographers (71%) selected the correct answer, namely that all the indicated factors were required to ensure optimal positioning of this projection (Figure 3). This difference observed between students and radiographers was statistically significant (*p* = 0.0099). However, 29% of radiographers only indicated that the hand should have been in supination, and 7% of students indicated that only the humeral epicondyles should be parallel to the imaging receptor (IR). Figure 4 demonstrates an AP (external rotation) shoulder image that adheres to the positioning criteria.

**FIGURE 5:**
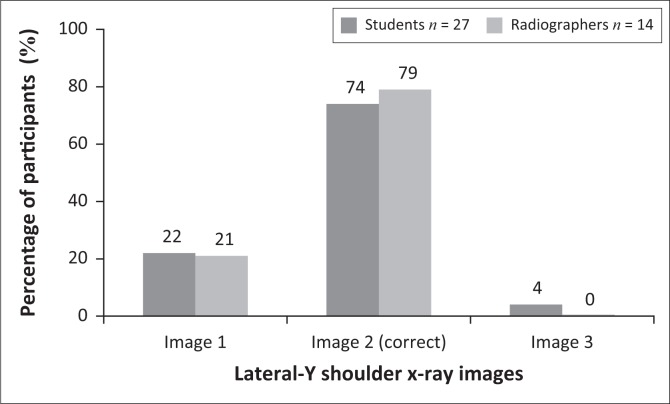
Participants’ answers with regard to the factors applicable to obtain optimal lateral-Y projection.

**FIGURE 6:**
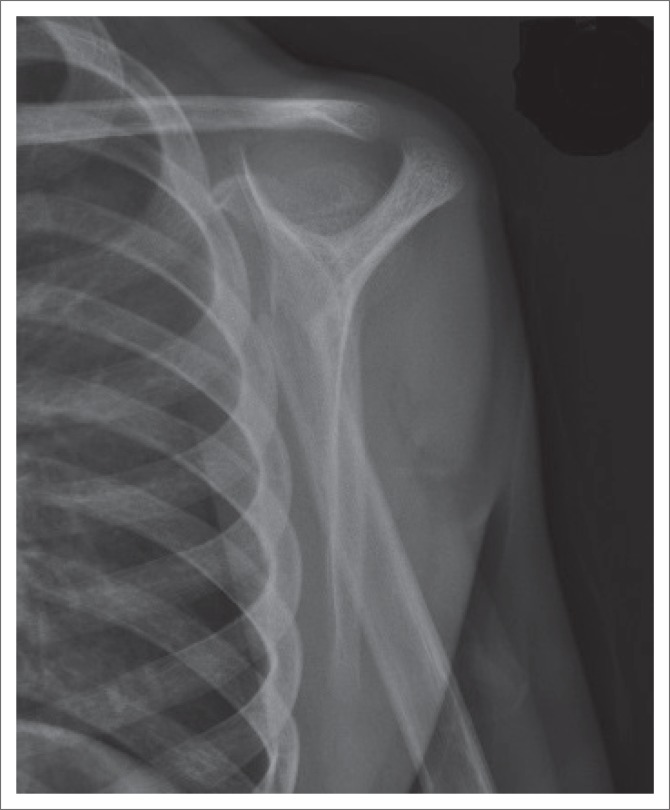
A lateral-Y shoulder image demonstrating correct positioning.

Three LAT-Y shoulder images were displayed and the participants had to identify the optimal shoulder image (Image 2) based on positioning and exposure factors. More than 70% of both the students and radiographers selected the correct image, as illustrated in Figure 5, with this difference not being significant (*p* = 1.0000). The correct positioning of a LAT-Y shoulder image is illustrated in Figure 6.

**FIGURE 7:**
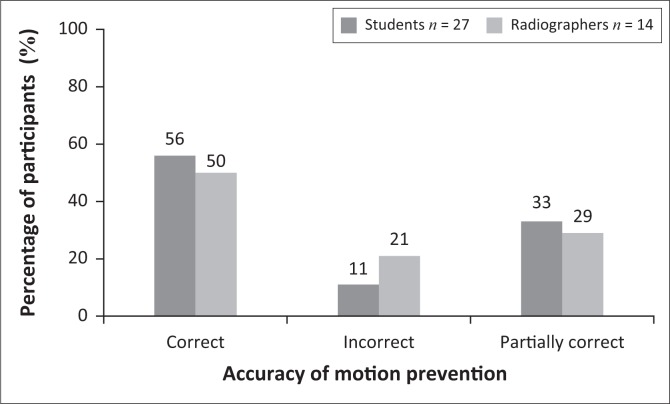
Participants’ answers to the question on measures applicable to prevent motion during imaging.

Participants were required to select the factors applicable to ensure that no motion occurred when obtaining X-ray projections of the shoulder. This question had two correct answers, namely applying a breathing technique and using a short exposure time. Figure 7 shows the results as correct, partially correct and incorrect. ‘Correct’ was recorded when the participant selected both answers (applying a breathing technique and using a short exposure time). ‘Partially correct’ meant that the participant had selected one of the two correct answers (applying a breathing technique or using a short exposure time), while ‘incorrect’ indicated that none of the correct answers was selected. With regard to this particular question, 11% of students and 21% of radiographers selected the incorrect answers, showing no significant difference between the two groups (*p* = 0.5157).

## Discussion

A large portion of the participants in this study was student radiographers (66%). Questions were based on the AP external rotation and LAT-Y shoulder projections, based on knowledge that the participants had acquired before in their theoretical training. The clicker test could be considered an effective method of administering the questionnaire because the participants immediately could see all the responses of their peers and reflect on their radiographic technique. Using the clickers did not cause the participants to experience any discomfort or distress, as the RCQ was administered anonymously.

### Selection of exposure

Milliamperage per second (mAs) is an important exposure factor that produces an X-ray image on which the bony structures and soft tissue can be visualised properly. In digital radiography, brightness/image density of an image on a display monitor is controlled by the window level and not the mAs. Less than 50% of radiographers identified the correct X-ray image demonstrating optimal mAs (Figure 1) for an AP projection (external rotation) of the shoulder, whereas 14% of radiographers selected the correct LAT-Y shoulder image demonstrating optimal mAs (Figure 2). With regard to the AP projection (external rotation), a significant difference was observed between students and radiographers. Only 52% of students identified the correct LAT-Y shoulder image demonstrating optimal mAs. It seems that the students, and more specifically the radiographers, could not identify optimal mAs on an X-ray image. It could be concluded that they did not realise or remember that mAs refers to the brightness/image density and that peak kilovoltage (kVp) refers to the grey scale present on an X-ray image. Hence, the participants struggled to assess brightness/image density on an image displayed on the monitor.

### Radiographic technique

Participants had to indicate the positioning factors that are of importance to ensure that an AP projection (external rotation) of the shoulder is optimal for diagnosis. These factors include some of the criteria applicable during imaging of the AP projection (external rotation) of the shoulder. It was noteworthy that most of the students indicated that all the factors, namely the hand in supination, arm abduction and the humeral epicondyles being parallel to the cassette, are of importance to obtain an optimal AP projection (external rotation) of the shoulder. A significant difference between the students and the radiographers were observed. It is clear that 29% of the radiographers did not know the positioning factors for the AP projection (external rotation), which could cause that these factors will be ignored during positioning, and consequently, the AP (external rotation) images would not adhere to the criteria outlined by the literature for an AP image of the shoulder. It is possible that the student radiographers answered this question correctly because they have obtained the theoretical training recently compared to the radiographers who completed their certificate or diploma or degree many years ago.

With regard to the participants’ knowledge of the correct breathing technique to apply when imaging the shoulder, more than 50% of radiographers and students indicated that applying a breathing technique and using a short exposure time would ensure that no motion occurs during imaging of the shoulder (Figure 7). A notable finding was that 29% of radiographers and 33% of students only indicated that a short exposure time is of importance to ensure the absence of motion, making no reference to the suspension of breathing (Figure 7). It is important to note that a breathing technique will contribute to reduce motion during imaging. It could therefore be concluded that for some of the students and radiographers, a breathing technique to prevent motion during imaging was overlooked or ignored.

The results of the study highlighted that the participants fail to apply some of the criteria as outlined by literature when evaluating routine shoulder projections. It is important to note that the significance difference was calculated not to compare the knowledge of students and radiographers against each other; however, it was to determine where and how necessary actions can be put in place to enhance the knowledge of the participants regarding the evaluation of shoulder images.

## Reliability and validity

Reliability was ensured through pilot-testing the technical aspects of the clicker questionnaire to maintain consistent scoring procedures by using the same RCQ under similar conditions (Delport & Roestenburg 2011).

Content validity entailed that all the content of the RCQ on the clicker system measured what it was intended to measure. The content was considered valid when based on a literature review and when it had been consulted with experts in the specific field of study (Brink et al. 2012; Delport & Roestenburg 2011; Institute for Work and Health 2007; Twycross & Shields 2004). In this case, the RCQ was compiled with published literature serving as a guideline, while the researcher also consulted experts in the field (in this case, experts in radiography and shoulder imaging) before collecting data, to ensure that the research instruments measured content validity. The use of a pilot study also increased the content validity of the study.

## Limitations

A limitation of the study was the presence of technical errors during the clicker session of the RCQ. These technical errors were because of failure to ensure that the correct answers were selected. After the student radiographers had completed the clicker session, the researcher realised that Question 37 did not select the correct answer in order for the TurningPoint program to calculate the percentages. It was corrected for the remaining clicker sessions, and the statistician manually calculated the percentages of the answers for this specific question as answered by the student radiographers.

## Recommendations

Because of the benefits and simplicity of using clickers to collect data, the use of this tool is recommended to facilitate the process of conducting research. The results are analysed as percentages, and the statistician can do further analysis as required for the specific study. It is suggested that the statistician analyse the data of the clicker session especially when more than one answer can be selected for a specific question.

Furthermore, clickers can also be utilised in radiography or any other profession for continuing professional development (CPD) activities to increase engagement and enhance knowledge and skills. The results illustrated that all the radiographers (qualified, supplementary, community service and student) are not evaluating the shoulder images correctly, and hence, it is recommended that the radiographic evaluation criteria are revised through CPD activities such as morning seminars. In-service training at the participating imaging department can also be arranged to enhance the radiographic technique of the radiographers.

## Conclusion

The knowledge (exposure factors and radiographic technique) of the participants could be investigated by means of the RCQ clicker system. Through the use of clickers, a gap in knowledge was identified, regarding exposure factors and the positioning criteria of the AP (external rotation) shoulder image that do exist among radiographers (qualified, supplementary, community service and student) when evaluating images of the shoulder. The use of clickers as a research method eased the research process for the researcher because of its benefits and simplicity. Further research is required to determine whether educators and researchers in other disciplines will consider the use of clickers to facilitate the process of collecting data for research.
